# The Epidemiology of UK Autoimmune Liver Disease Varies With Geographic Latitude

**DOI:** 10.1016/j.cgh.2021.01.029

**Published:** 2021-12

**Authors:** Gwilym J. Webb, Ronan P. Ryan, Tom P. Marshall, Gideon M. Hirschfield

**Affiliations:** ∗ National Institute for Health Research, Birmingham Biomedical Research Centre, Birmingham, United Kingdom; § Primary Care Clinical Sciences, Institute of Applied Health Research, University of Birmingham, Birmingham, United Kingdom; ‡ Cambridge Liver Unit, Addenbrooke’s Hospital, Cambridge, United Kingdom; || Division of Gastroenterology and Hepatology, Toronto Centre for Liver Disease, University of Toronto, Toronto, Ontario, Canada

**Keywords:** Autoimmune Liver Disease, Latitude, Primary Biliary Cholangitis, Primary Sclerosing Cholangitis, Autoimmune Hepatitis, AIH, autoimmune hepatitis, AILD, autoimmune liver disease, IQR, interquartile range, MS, multiple sclerosis, PBC, primary biliary cholangitis, PSC, primary sclerosing cholangitis, THIN, The Health Improvement Network

## Abstract

**Background & Aims:**

The epidemiology of autoimmune liver disease (AILD) is challenging to study because of the diseases’ rarity and because of cohort selection bias. Increased incidence farther from the Equator has been reported for multiple sclerosis, another autoimmune disease. We assessed the incidence of primary biliary cholangitis (PBC), primary sclerosing cholangitis (PSC), and autoimmune hepatitis (AIH) in relation to latitude.

**Methods:**

We performed a retrospective cohort study using anonymized UK primary care records from January 1, 2002, to May 10, 2016. All adults without a baseline diagnosis of AILD were included and followed up until the first occurrence of an AILD diagnosis, death, or they left the database. Latitude was measured as registered general practice rounded down to whole degrees.

**Results:**

The cohort included 8,590,421 records with 53.3 × 10^7^ years of follow-up evaluation from 694 practices. There were 1314 incident cases of PBC, 396 of PSC, and 1034 of AIH. Crude incidences were as follows: PBC, 2.47 (95% CI, 2.34–2.60); PSC, 0.74 (95% CI, 0.67–0.82); and AIH, 1.94 (95% CI, 1.83–2.06) per 100,000 per year. PBC incidence correlated with female sex, smoking, and deprivation; PSC incidence correlated with male sex and non-smoking; AIH incidence correlated with female sex and deprivation. A more northerly latitude was associated strongly with incidence of PBC: 2.16 (95% CI, 1.79–2.60) to 4.86 (95% CI, 3.93–6.00) from 50°N to 57°N (*P* = .002) and incidence of AIH: 2.00 (95% CI, 1.65–2.43) to 3.28 (95% CI, 2.53–4.24) (*P* = .003), but not incidence of PSC: 0.82 (95% CI, 0.60–1.11) to 1.02 (95% CI, 0.64–1.61) (*P* = .473). Incidence after adjustment for age, sex, smoking, and deprivation status showed similar positive correlations for PBC and AIH with latitude, but not PSC. Incident AIH cases were younger at more northerly latitude.

**Conclusions:**

We describe an association in the United Kingdom between more northerly latitude and the incidence of PBC and AIH that requires both confirmation and explanation.

**What You Need to Know**

**Background**

Disease risk in primary biliary cholangitis, primary sclerosing cholangitis, and autoimmune hepatitis is insufficiently explained by genetic factors. In other autoimmune diseases such as multiple sclerosis, geographic latitude correlates with disease risk.

**Findings**

We show an association between more northerly geographic latitude and disease incidence of primary biliary cholangitis and autoimmune hepatitis, but not primary sclerosing cholangitis, in a large UK primary care population.

**Implications for patient care**

Although these findings require confirmation in other populations, they inform further exploration of pathogenic mechanisms influencing disease risk such as vitamin D deficiency.

The three major autoimmune liver diseases (AILDs), primary biliary cholangitis (PBC), primary sclerosing cholangitis (PSC), and autoimmune hepatitis (AIH), represent significant causes of liver morbidity and mortality whose etiopathogenesis is incompletely understood.^1^, ^2^, ^3^ Although individually uncommon, together they account for a significant proportion of elective liver transplantation,^4^ and the incidence and prevalence appear to be increasing.^5^, ^6^, ^7^, ^8^

To date, genetic association studies only have been able to explain a minority of the risk for the three AILDs, suggesting a significant role for environmental factors.^9^ A number of environmental factors have been identified as associated with risk of AILD including urinary tract infections, nail polish use, hair dye use, smoking, and deprivation in PBC^10^, ^11^, ^12^, ^13^, ^14^; smoking as a negative risk factor in PSC^15^^,^^16^; and various drug exposures in AIH.^3^^,^^17^^,^^18^ However, even in aggregate, these factors appear insufficient to explain variations in individual disease risk.^9^

Latitude has been proposed as a risk factor for AILDs because of its link to vitamin D status and in turn the link between vitamin D status and risk of autoimmunity.^19^, ^20^, ^21^, ^22^, ^23^ Type 1 T helper (T_H_1) T-cell activation and B-cell activation are implicated in each of the AILDs, and activation is regulated negatively by physiological concentrations of vitamin D.^1^, ^2^, ^3^^,^^22^ The association between the neurologic autoimmune disease multiple sclerosis (MS) and latitude is well established.^24^^,^^25^ For type 1 diabetes, childhood hypovitaminosis D has been associated with increased disease risk.^26^ By analogy, similar associations with latitude exposure have been shown in other autoimmune conditions and latitude has been suggested as a target for investigation among the AILDs, but to date such an assessment has not been performed.^21^^,^^27^

In this study we investigated the relationship between latitude and incidence of AILD in a large UK primary care database with established generalizability to the national population.^28^ We also describe disease prevalence and assess disease associations with age, sex, deprivation, smoking, and ethnicity.

## Methods

### Study Design and Population

A retrospective cohort study was performed using the pseudonymized primary care health records contained in The Health Improvement Network (THIN) database between January 1, 2002, and May 10, 2016.^29^ THIN is a UK-based primary care database containing routinely collected electronic patient records. At each consultation, general practitioners record details of the medical encounter, including diagnosis. Demographic details such as age, sex, and linked deprivation scores also form part of the electronic record. Deprivation is based on a combination of scores for home ownership, car ownership, unemployment, and household overcrowding, and is adjusted so that the country is represented as 5 equally sized quintiles as originally described by Townsend^30^ in 1987.

### Inclusion Criteria

Patients of all ages registered with a practice contributing to THIN during the study period.

### Exclusion Criteria

Patients with a previous diagnosis of AILD at baseline were excluded. Patients with potential overlap of autoimmune liver diseases were excluded (see later).

### Follow-Up Evaluation

Patients were followed up until death, leaving a contributing practice, the date that a practice stopped contributing to THIN, or the end of the study period, whichever was latest.

### Exposure

Latitude was measured by geocoding latitude from the postal code of the general practice concerned. This was performed by THIN to avoid identification of individual practices; the locations of individual practices were not available to the authors. Latitude was rounded down to the nearest integer. Because of the small population residing in the 58° latitude band, the residents at 58° were combined with the residents at 57° for analysis. Townsend^30^ quintiles were calculated from the postcode of place of residence by THIN. Covariable data on age, sex, smoking status, and ethnicity were collected from general practice records. The most recently submitted data in each category at the end of the follow-up period were used in each case.

### Outcomes

Diagnoses of AILDs were defined using diagnostic codes (Supplementary Table 1). For PBC and PSC, a single diagnostic code specific to the respective condition was identified; for AIH, a combination of 3 potential codes validated in a previous study was used.^31^ Of note, in contrast to the International Classification of Diseases system of diagnostic codes, the Read Code system used in UK general practice contains a specific term for PSC. For use as positive and negative controls of conditions with and without published associations with latitude, cases of MS and hypertension were examined using standard diagnostic codes (Supplementary Table 1).

Cases with 2 or more lifetime diagnoses of different AILDs (ie, cases of possible autoimmune overlap) were not considered to represent incident disease for any AILD diagnosis. Cases were considered incident if the date of their first recording was more than 1 year after the individual’s registration at the practice concerned, and also 1 year after the practice had achieved acceptable mortality reporting.^32^ This was to prevent pre-existing diagnoses appearing incident in new registrations at a particular general practice.^33^

Data regarding the Townsend^30^ deprivation quintile were provided by THIN and derived from the postal code of the patient’s residence. Latitude bands were provided by THIN at special request and were derived from the practice postal code.

All data used were those most recent at the end of the follow-up period for a given individual.

### Statistical Analyses

All data were analyzed using Stata v15.1 (StataCorp, College Station, TX) using the University of Birmingham BlueBEAR high performance computer cluster. We present descriptive statistics, univariable analysis of associations between risk factors and incidence, and multivariable analysis with adjustments for sex, age, smoking status, Townsend^30^ deprivation quintile, and latitude. Where data were adjusted for covariables, direct standardization was used. Where direct standardization was used to adjust for multiple covariables, individuals with missing data in any category were excluded. For the examination of trends over time, latitude, or quintiles of deprivation, a least-squares linear regression was performed. For assessing for changes in sex ratios, the chi-squared test for trend was used.

Maps were produced using QGIS v3.49 (https://www.qgis.org) using public domain shapefiles from the UK Ordnance Survey OpenData project.

### Ethical Approval

THIN previously received approval for research using its database from the National Health Service South-East Multi-Centre Research Ethics Committee in 2003. This study received approval from the THIN Scientific Review Committee (reference 16THIN055).

## Results

Overall, a total of 8,590,421 pseudonymized patient records with a total of approximately 53.3 million years of follow-up evaluation from 694 practices were examined. The first entry to follow-up was January 1, 2002, and the last data collection point was May 10, 2016. The median follow-up period was 5.2 years (interquartile range [IQR], 1.9–10.2 y). A total of 1314 incident cases of PBC, 396 incident cases of PSC, and 1034 incident cases of AIH were identified.

### Incidence and Prevalence

The summary details of incident cases are provided in Supplementary Tables 1 and 2. When assessed by linear regression, there was no significant change in the incidence of any of PBC, PSC, or AIH over the study period (Supplementary Table 3 and Supplementary Figure 1).

In 2015, the last full year of the study, a total of 1299 prevalent cases of PBC, 353 cases of PSC, and 1116 cases of AIH were identified. Details of disease prevalence are summarized in Table 2 and Supplementary Table 4. The prevalence of all 3 diseases increased over time (Supplementary Figure 2). Of those prevalent cases of PSC in 2015, 52% had a lifetime diagnosis of ulcerative colitis, and 15% a lifetime diagnosis of Crohn’s disease.

### Latitude

To confirm whether a previously reported correlation of incidence with latitude could be shown using this data set, the incidence of MS was assessed. The overall crude incidence of MS was 8.98 (8.75–9.23) per 100,000/y.

When assessed by latitude band after adjustment for sex, age, smoking status, and Townsend^30^ quintile, there was a higher incidence of MS in the in the 57° band at 13.67 (11.86–15.48) per 100,000/y than in the 50° latitude band at 8.24 (7.40–9.07) per 100,000/y (Supplementary Figure 3 and Supplementary Table 5). There was an increase in MS incidence of 0.66 (0.25–1.07) per 100,000/y per degree in latitude increase (r^2^ = 0.721; *P* = .008). By contrast, for hypertension, a disease not reported to have an association with latitude, overall incidence was 946.26 (943.58–948.94) per 100,000/y with no significant correlation with latitude at -5.02 (-30.27 to 20.23) per 100,000/y per degree in latitude (r^2^ = 0.038; *P* = .644) (Supplementary Figure 3 and Supplementary Table 5).

**Figure 1:**
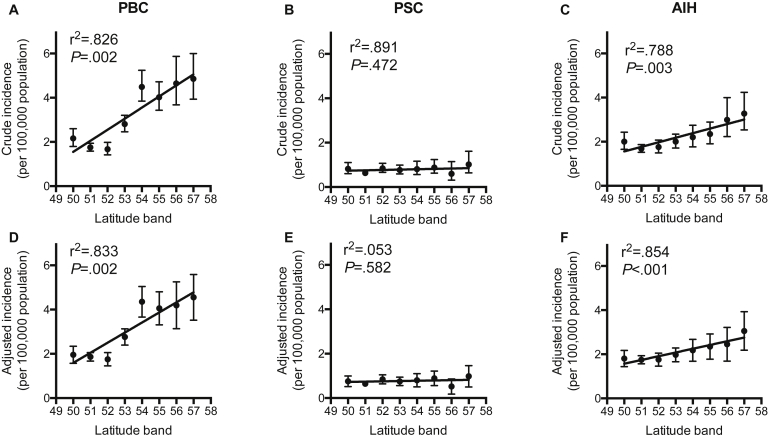
Incidence of autoimmune liver disease by latitude. *Top row*: Plots denote crude incidence of (*A*) primary biliary cholangitis (PBC), (*B*) primary sclerosing cholangitis (PSC), and (*C*) autoimmune hepatitis (AIH). (*D–F*) *Bottom row*: Plots denote adjusted incidence after adjustment for sex, age, smoking status, and Townsend^30^ deprivation quintile. For PBC and AIH there was a significant increase in incidence at more northerly latitudes both before and after adjustment; for PSC a significant correlation was not present.

**Figure 2:**
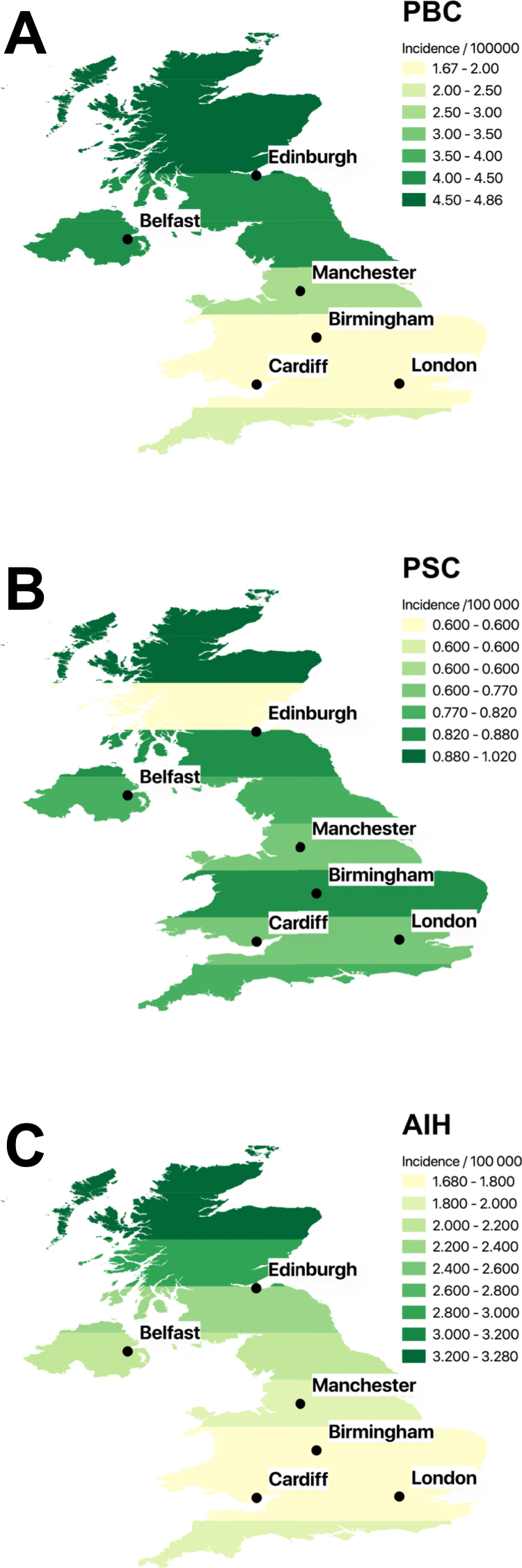
Maps of the United Kingdom showing the crude incidence of autoimmune liver diseases. Incidences are for the whole study period and shown as cases per 100,000/y. The density of shading corresponds to incidence as denoted in each panel: (*A*) primary biliary cholangitis (PBC), (*B*) primary sclerosing cholangitis (PSC), and (*C*) autoimmune hepatitis (AIH). The locations of major cities are shown for reference.

For AILDs, the crude incidence of PBC was markedly greater at more northerly latitudes (Table 1, Supplementary Table 2, Figure 1, Figure 2, and Supplementary Figure 4). After adjustment for sex, age, smoking status, and Townsend^30^ deprivation quintile, there remained a more than doubling in incidence from the 1.96 (1.57–2.34) per 100,000/y in the 50° latitude band to 4.55 (3.52–5.58) per 100,000/y in the 57° latitude band. When assessed by linear regression, PBC incidence increased by 0.46 (0.27–0.66) per 100,000/y per degree in latitude (r^2^ = 0.850; *P* = .001). Similarly, but less markedly, the incidence of AIH was greater at more northerly latitudes at 0.19 (0.11–0.26) per 100,000/y per degree (r^2^ = 0.873; *P* < .001). PSC incidence showed no significant correlation with latitude at 0.01 (-0.02 to 0.04) per 100,000/y (r^2^ = 0.055; *P* = .577).

**Table 1:** Incidence per 100,000 per Year of PBC, PSC, and AIH for the Time Period January 1, 2002 to May 10, 2016

Category	Subcategory	PBC			PSC			AIH		
		n	Incidence	95% CI	n	Incidence	95% CI	n	Incidence	95% CI
Population	–	1314	2.47	2.33–2.60	396	0.74	0.67–0.82	1034	1.95	1.83–2.07
Latitude	50°-	111	1.96	1.57–2.34	42	0.76	0.52–1.00	103	1.80	1.44–2.17
	51°-	373	1.86	1.67–2.06	135	0.65	0.54–0.75	359	1.75	1.57–1.93
	52°-	135	1.75	1.45–2.05	68	0.84	0.64–1.05	142	1.76	1.46–2.05
	53°-	220	2.76	2.40–3.13	60	0.75	0.56–0.94	157	1.97	1.66–2.28
	54°-	161	4.35	3.66–5.05	29	0.80	0.50–1.10	79	2.18	1.68–2.67
	55°-	151	4.06	3.31–4.80	33	0.89	0.56–1.22	88	2.35	1.77–2.92
	56°-	70	4.19	3.13–5.25	9	0.52	0.18–0.86	45	2.45	1.69–3.22
	57°-	86	4.55	3.52–5.58	18	0.98	0.50–1.46	58	3.06	2.18–3.93
	Missing	7	2.36	0.57–4.15	2	0.56	0.00–1.33	3	0.73	0.00–1.57
Sex	Male	173	0.66	0.56–0.76	230	0.91	0.79–1.03	241	0.94	0.82–1.06
	Female	1141	4.24	3.99–4.49	166	0.59	0.50–0.68	793	2.92	2.72–3.13
Age, *y*	0–9.9	0	0.00	0.00–0.00	2	0.00	0.00–0.00	7	0.12	0.00–0.26
	10–19.9	1	0.01	0.00–0.02	21	0.03	0.01–0.04	55	0.93	0.54–1.32
	20–29.9	5	0.07	0.01–0.13	34	0.05	0.03–0.07	48	0.62	0.44–0.81
	30–39.9	59	0.71	0.52–0.90	28	0.03	0.02–0.05	87	1.10	0.87–1.34
	40–49.9	181	1.94	1.65–2.23	68	0.08	0.06–0.10	132	1.45	1.20–1.70
	50–59.9	318	4.10	3.52–4.68	61	0.08	0.06–0.10	221	2.70	2.34–3.07
	60–69.9	353	5.08	4.53–5.63	87	0.14	0.10–0.17	239	3.68	3.13–4.23
	70–79.9	277	6.22	5.20–7.25	65	0.15	0.10–0.19	181	3.72	3.15–4.29
	80–89.9	111	4.28	3.20–5.36	28	0.09	0.05–0.13	61	2.34	1.67–3.00
	≥90	9	1.21	0.38–2.05	2	0.02	0.00–0.05	3	0.71	0.00–1.65
Deprivation	1 (least)	268	2.21	1.91–2.51	99	0.67	0.53–0.81	242	1.85	1.59–2.11
	2	287	2.43	2.14–2.72	88	0.74	0.58–0.91	225	1.90	1.65–2.16
	3	282	2.60	2.29–2.91	87	0.81	0.64–0.98	219	2.02	1.75–2.29
	4	244	2.62	2.28–2.96	69	0.80	0.61–1.00	175	1.95	1.65–2.25
	5 (most)	187	2.82	2.35–3.28	39	0.73	0.46–0.99	141	2.25	1.82–2.67
	Missing	46	2.86	1.93–3.79	14	0.75	0.35–1.15	32	1.76	1.06–2.45
Smoking	Smoker	400	3.40	3.03–3.77	54	0.47	0.33–0.61	256	2.24	1.94–2.55
	Ex-smoker	308	2.93	2.58–3.27	69	0.61	0.45–0.77	185	1.98	1.54–2.42
	Never smoker	596	1.96	1.80–2.12	265	0.95	0.83–1.07	578	1.96	1.79–2.12
	Missing	10	0.87	0.17–1.57	8	0.26	0.00–0.56	15	0.30	0.00–0.64

NOTE. Figures are adjusted for latitude, sex, age, Townsend^30^ deprivation quintile, and smoking status by direct standardization as appropriate. Figures are per 100,000 population/y with 95% CIs. Deprivation refers to Townsend^30^ deprivation quintiles. See also Supplementary Table 2 for unadjusted figures.

AIH, autoimmune hepatitis; PBC, primary biliary cholangitis; PSC, primary sclerosing cholangitis; CI, confidence interval.

**Table 2:** Prevalence of PBC, PSC, and AIH in 2015

Category	Subcategory	PBC			PSC			AIH		
		n	Prevalence	95% CI	n	Prevalence	95% CI	n	Prevalence	95% CI
Population	–	1299	39.62	37.50–41.74	353	10.77	9.65–11.88	1116	34.04	32.06–36.02
Latitude	50°-	83	28.16	21.88–34.44	27	9.71	5.87–13.55	93	32.19	25.38–39.00
	51°-	381	30.59	27.50–33.69	141	10.61	8.84–12.38	382	29.78	26.76–32.79
	52°-	84	27.29	21.44–33.15	30	9.90	6.29–13.50	88	29.00	22.89–35.10
	53°-	161	42.01	35.51–48.51	34	8.55	5.64–11.45	143	37.20	31.04–43.37
	54°-	153	63.86	53.70–74.02	27	11.41	7.09–15.74	98	42.69	34.10–51.30
	55°-	195	49.79	42.02–57.56	40	10.46	7.01–13.91	148	40.47	33.02–47.93
	56°-	103	60.95	48.53–73.36	18	10.63	5.67–15.58	71	41.00	31.17–50.82
	57°-	129	61.08	50.11–72.05	34	16.70	10.77–22.63	88	39.11	30.44–47.78
	Missing	10	37.40	15.06–59.74	2	8.51	0.00–19.85	5	18.50	3.98–33.03
Sex	Male	143	8.82	7.37–10.28	205	13.25	11.43–15.07	283	17.93	15.83–20.04
	Female	1156	69.35	65.36–73.33	148	8.69	7.28–10.09	833	50.36	46.92–53.79
Age	0-9.9	0	0.00	0.00–0.00	1	0.06	0.00–0.18)	2	2.55	0.00–7.43
	10-19.9	0	0.00	0.00–0.00	11	3.64	1.40–5.89	28	9.02	4.34–13.68
	20-29.9	2	0.77	0.00–1.95	33	7.23	4.14–10.32	67	16.74	10.76–22.71
	30-39.9	31	5.88	3.78–7.98	42	8.30	5.72–10.88	82	23.58	14.76–32.40
	40-49.9	121	21.04	17.27–24.82	44	9.09	5.44–12.73	132	28.08	18.51–37.65
	50-59.9	260	52.81	41.85–63.77	67	12.34	9.36–15.32	201	35.66	30.68–40.65
	60-69.9	363	84.44	70.06–98.82	73	15.81	12.07–19.55	279	68.96	55.14–82.77
	70-79.9	344	100.97	89.93–112.02	54	19.14	12.56–25.72	221	66.06	56.93–75.20
	80-89.9	163	84.97	70.48–99.46	26	12.41	7.34–17.49	97	57.29	44.49–70.10
	90+	15	29.64	13.90–45.37	2	3.38	0.00–7.90	7	33.83	0.96–66.69
Deprivation	1 – least	269	38.15	32.99–43.30	13	9.84	6.14–13.53	233	31.40	26.79–36.00
	2	295	43.15	38.50–48.36	88	11.92	9.67–14.69	261	35.87	31.40–40.34
	3	268	39.24	34.82–44.24	83	12.14	9.79–15.05	247	36.30	31.77–40.83
	4	256	41.82	37.00–47.27	70	10.25	8.11–12.96	189	32.09	27.41–36.78
	5 – most	175	40.28	34.74–46.72	64	10.45	8.18–13.36	156	37.31	30.60–44.00
	Missing	36	28.32	20.43–39.27	35	8.06	5.78–11.22	30	27.27	16.52–38.03
Smoking	Smoker	347	48.60	43.04–4.15	41	5.27	3.54–7.00	273	40.01	34.37–45.66
	Ex-smoker	351	52.55	46.96–58.13	60	8.49	6.19–10.80	234	36.33	31.46–41.21
	Never smoker	596	31.43	28.89–33.97	247	14.13	12.34–15.93	595	32.73	29.91–35.57
	Missing	5	10.69	0.61–20.77	5	3.92	0.00–8.14	14	23.31	8.09–38.55

NOTE. Figures are adjusted for latitude, sex, age, Townsend^30^ deprivation quintile, and smoking status by direct standardization as appropriate. Figures are per 100,000 population with 95% CIs. Deprivation refers to Townsend^30^ deprivation quintiles. See also Supplementary Table 4 for unadjusted figures.

AIH, autoimmune hepatitis; PBC, primary biliary cholangitis; PSC, primary sclerosing cholangitis; CI, confidence interval.

**Figure 3:**
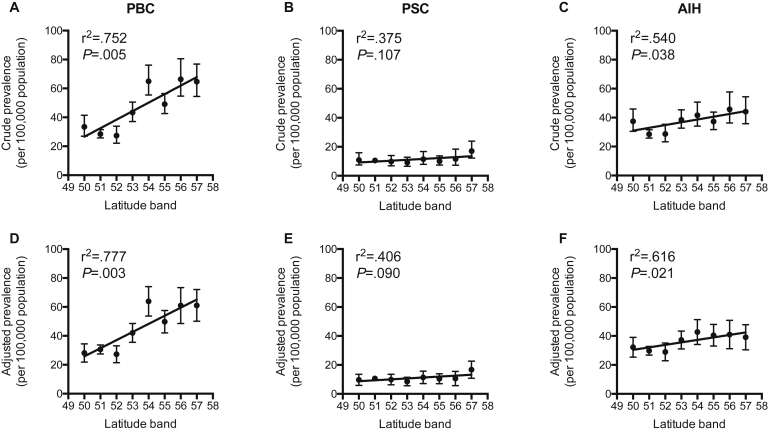
Prevalence of autoimmune liver disease by latitude. *Top row*: Plots denote crude prevalence at the end of 2015 of (*A*) primary biliary cholangitis (PBC), (*B*) primary sclerosing cholangitis (PSC), and (*C*) autoimmune hepatitis (AIH). (*D–F*) *Bottom row*: Plots denote adjusted incidence after adjustment for sex, age, smoking status, and Townsend^30^ deprivation quintile. For PBC, 5.89 (2.51–9.27) per 100,000 per degree (r^2^ = 0.752; *P* = .005); PSC, 0.62 (-0.18 to 1.41) per 100,000 per degree (r^2^ = 0.375; *P* = .107); and AIH, 1.92 (0.150–3.69) per 100,000 per degree (r^2^ = 0.540; *P* = .038).

In 2015, after adjustment for age, sex, smoking status, and Townsend^30^ quintile, there was a significant increase in the prevalence of both PBC and AIH at more northerly latitudes; such a gradient was not apparent for PSC (Table 2, Supplementary Table 4, and Figure 3). The prevalence of PBC increased by 5.61 (2.61–8.62) per 100,000 per degree of latitude (r^2^ = 0.777, *P* = .003). For PSC, the prevalence did not change significantly at 0.64 (-0.14 to 1.40) per 100,000 per degree of latitude (r^2^ = 0.406, *P* = .090). For AIH, the prevalence increased by 1.72 (0.36–3.08) per 100,000 per degree of latitude (r^2^ = 0.616, *P* = .021).

Given the apparent differences in incidence between latitude bands, patient sex and age at presentation were assessed by latitude band. There was a significant trend toward the younger incidence of AIH at more northerly latitudes (-0.87; -1.49 to -0.25) years per degree (r^2^ = 0.663, *P* = .014), but no significant difference in age for incident PBC or PSC, and no significant difference in incident sex ratios in any disease (Supplementary Tables 6 and 7).

### Sex and Age

Over the whole study period, 1141 (86.8%) incident cases of PBC were female, with a female:male ratio of 6.6:1; for PSC, 230 (58.1%) were male, with a 1.4:1 male:female ratio; for AIH, 793 (76.6%) were female, with a female:male ratio of 3.3:1. There was no significant change in the sex ratio of incident cases over time for any of the conditions (Supplementary Table 8). The median age of incident PBC was 63 years (IQR, 53–72 y); for PSC the median age was 57 years (IQR, 43–69 y); and for AIH the median age was 58 years (IQR, 44–69 y). The age of incidence did not change for PBC and PSC over the study period, but for AIH the median age at incidence increased (Supplementary Table 9).

### Smoking

After adjustment for sex, age, Townsend^30^ deprivation quintile, and latitude, incident PBC was more frequent in smokers than those who had never smoked at 3.40 (3.03–3.77) per 100,000/y and 1.96 (1.80–2.12) cases per 100,000/y, respectively. After the same adjustments, there was a lower incidence of PSC in smokers 0.47 (0.33–0.61) per 100,000/y compared with those who had never smoked 0.95 (0.83–1.07) per 100,000/y. For AIH, there was no difference between current smokers and those who had never smoked.

### Deprivation

PBC was associated significantly with deprivation. The incidence was 2.21 (1.91–2.51) per 100,000/y, and in the most deprived quintile the incidence was 2.82 (2.35–3.28) per 100,000/y after adjustment for age, sex, latitude, and smoking status. When assessed by linear regression, there was an increase in incidence of 0.14 (0.08–0.20) per 100,000/y per Townsend^30^ quintile (r^2^ = 0.946; *P* = .005). The incidence of PSC and AIH did not vary significantly with deprivation; deprivation varied with latitude (Supplementary Table 10)

### Ethnicity

Data on ethnicity were missing for 5,008,804 (58.3%) registrations and a similar proportion of those diagnosed with AILD (Supplementary Tables 2 and 4). Among the minority with a recorded ethnicity, the crude incidence and prevalence of PBC among those of white ethnicity was greater than in those of non-white ethnicities. For PSC and AIH, there were no significant differences. Attempts to adjust for covariables were not made because of the high frequency of missing data.

## Discussion

In this study we used aggregate data from a nationally representative cross-section of primary care practices to show markedly greater incidences of both PBC and AIH at more northerly latitudes. For PBC there was a greater than doubling of incidence over 7º of latitude; for AIH there was an increase of more than 50%. These observations persisted after correction for sex, age, deprivation, and smoking status. For PBC, the difference was notable for being more marked than the fold change in incidence for MS in either this study or in meta-analyses.^24^^,^^25^

One potential explanation for a disease correlation with latitude is varying sunlight (ultraviolet light) exposure and its effects on vitamin D metabolism. Such a pathway has been proposed in PBC previously.^21^^,^^34^ However, if this were a major etiologic factor, patients with more pigmented skin might be expected to be at increased risk.^35^ Perhaps differences in sunlight exposure modulate genetic risk. In addition, it is unclear as to what stage in life any exposure effect from differing latitudes may occur and it is plausible that it is childhood exposure that is most important.^26^ Expanding this work to other geographic areas is necessary. Equally, looking for similar correlations in disease incidence and latitude in other diseases associated with low vitamin D status such as inflammatory bowel disease would be valuable.^36^ In addition, environmental factors other than vitamin D may be important. Smoking correlates with both PBC and AIH incidence and it may be that our study has undercorrected for these. Other environmental toxins that have been proposed include toxic waste, coal, and hair dye.^10^^,^^37^^,^^38^ It is unknown as to how these might correlate with latitude. It is equally possible that some previously unconsidered factor perhaps relating to geology, diet, local flora, or air quality is responsible for these results. In addition, the genetic make-up of the United Kingdom varies significantly with geography and it may be that our results reflect an unrecognized genetic tendency.^39^

Two other observations within our results warrant discussion in the context of others’ work. First, we have shown no correlation between PSC and latitude, whereas a high prevalence of PSC has been reported in the Nordic countries, which are at northern latitudes.^40^ Some of this risk, however, may be genetic.^41^ In addition, the Nordic countries have low rates of smoking and are prosperous. Notably, studies from Canada, which is also at a relatively northerly latitude, do not record disproportionately high rates of PSC.^5^ Second, we recorded a lower median age at presentation among patients with AIH at more northern latitudes in our cohort. Although this may correlate with exposure to an unknown environmental factor, it also may relate to genetic predisposition in the context of known marked geographic genetic variations in the United Kingdom.^39^ Variations in age of presentation in relation to HLA-D haplotype are well recognized in AIH, as are variations in age of presentation internationally.^3^ To consider both questions more fully, additional studies with consistent approaches to diagnosis based over large and varying geographic areas are required.

Our estimates for incidence are close to those reported elsewhere: in a recent meta-analysis, the population incidence of PSC across a number of countries was estimated at 0 to 1.3 per 100,000/y, and at 0.33 to 5.8 per 100,000/y for PBC.^5^ By comparison, in this study the overall incidence was calculated at 0.74 per 100 000/y and 2.47 per 100,000/y, respectively. For AIH, meta-analyses are lacking, but 2 recent studies from Europe reported incidences of 1.1 per 100,000/y and 1.7 per 100,000/y; we report an incidence of 1.94 per 100,000/y.^6^^,^^7^ Our overall prevalence estimates also are similar to those published elsewhere.

There are likely to be differences in this cohort compared with others. We reported median ages for the diagnosis of AILD that are higher than those reported elsewhere. For example, the median age at diagnosis of PBC in our cohort was 63 years; in the UK-PBC national cohort the median age was 55 years.^42^ For PSC, our median age was 57 years, the International PSC Study Group reported a mean age at diagnosis of 39 years.^43^ Such differences may reflect age-associated differences in referral patterns to, or retention of follow-up evaluation in, secondary care; bias in entry into registries; or differences in diagnostic classification between primary and secondary care. Furthermore, our work primarily examined adults, whereas the International PSC Study Group cohort, for example, contained 13% of individuals aged younger than 20. Our work has shown an apparent narrowing in the female:male ratio of patients referred for transplantation in PBC.^4^ In this study, there was no change over time in the sex ratio for any disease including PBC. This may reflect the reported poorer outcome for men than women diagnosed with PBC, an observation that in turn has been related to later diagnosis.^42^

In this study we confirm the previously identified dichotomous effect of smoking on AILD risk by showing an association with increased incidence of PBC, but with decreased PSC risk. Notably, these associations persisted after controlling for deprivation, which may be associated with smoking behavior.^44^ We note negative national trends in smoking over our study period, although this study was not powered to show differences in the duration of exposure and time from exposure that might be expected to affect future AILD epidemiology as national smoking trends change.^45^

Ethnicity, with its potential associations with both genetics and environment, remains an incompletely understood component of AILD risk. Interpreting the role of ethnicity and disease risk in this study was hampered by a large proportion of individuals not having a recorded ethnicity and there being relatively few patients of non-white ethnicities. However, we do describe an approximately doubled incidence of PBC in those of white ethnicity as compared with those of other ethnicities. Such an association has been described elsewhere, but this large cohort underlines such differences.^46^ We are unable to comment on differences in the likelihood of investigation for PBC in these populations or on disease trajectory or severity at diagnosis described by others.^47^

This study has several strengths: its 14-year time frame, large cohort size, the presence of information about co-factors, and its derivation from primary care records rather than secondary care records avoided the inherent risk of selection bias in the latter. Potential weaknesses of this study include the possibility of inaccurate recording of diagnoses. We note, however, work suggesting that at least for AIH, primary care Read Codes are broadly representative of specialist diagnoses.^31^ Furthermore, although we have corrected for a major measure of socioeconomic grouping by using Townsend^30^ quintiles, we may have not corrected sufficiently and some of the gradient in incidence we are seeing may reflect factors associated with this such as dietary habits, exposure to environmental toxins, or access to health care. In addition, we may have underestimated the incidence of these diseases because of our exclusion of overlapping disease and incident disease within 1 year of joining a general practice. We cannot exclude the existence of an alternative confounding factor in relation to genetics, the environment, or medical practice that would explain the variations in PBC and AIH seen at different latitudes. We note specifically the existence of a center with a particular focus on AILDs in Newcastle in the 54° latitude band. Our findings require confirmation in a different geographic region. A high proportion of individuals did not have ethnicity recorded, meaning that this was excluded from analysis and ethnicity represents a further potential confounder. Finally, we were unable to account for previous places of residence for individuals who had changed location over time.

Here, we show a striking correlation between geographic latitude and disease risk for PBC; the same phenomenon is present to a lesser extent for AIH, but absent for PSC. We also present key demographic information with regards to sex, age, deprivation, and smoking status derived from primary care data. Our results support a new avenue of investigation in AILD etiology, provide primary care–derived estimates of AILD epidemiology, and confirm others’ findings regarding the environmental impact of smoking, deprivation, and ethnicity on the AILDs.
