# Supplementary material for: The Epidemiology of UK Autoimmune Liver Disease Varies With Geographic Latitude
Source: Clin Gastroenterol Hepatol. 2021 Dec;19(12):2587–96. doi: 10.1016/j.cgh.2021.01.029 (PMC8661127; doi:10.1016/j.cgh.2021.01.029)
Supplement: Supplementary Material S2 [file mmc1.pdf]

The epidemiology of UK autoimmune liver disease varies with geographic latitude

Gwilym J Webb[1,2], Ronan P Ryan[3], Tom P Marshall[3], and Gideon M Hirschfield[1,4]

[1]National Institute for Health Research (NIHR) Birmingham Biomedical Research Centre (BRC), University of Birmingham, UK

[2]Cambridge Liver Unit, Addenbrooke's Hospital, Cambridge, UK

[3]Primary Care Clinical Sciences, Institute of Applied Health Research, University of Birmingham, UK

[4]Toronto Centre for Liver Disease, Ontario, Canada

Supplementary figures

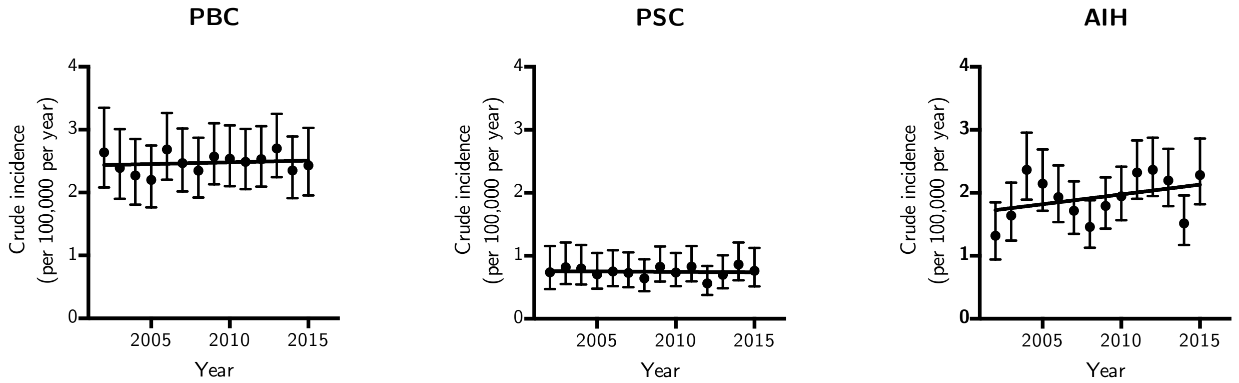

Figure S1: Crude incidence per 100 000 per year of autoimmune liver disease over the study period. There was no significant change for any of the diseases over the study period. For PBC, 0.01(-0.02 to 0.03)/100 000/year/year; r^2=0.023; p=0.693. For PSC, 0.00(-0.01 to 0.01)/100 000/year/year; r^2=0.004; p=0.837. For AIH, 0.03(-0.02 to 0.08)/100 000/year/year; r^2=0.130; p=0.205.

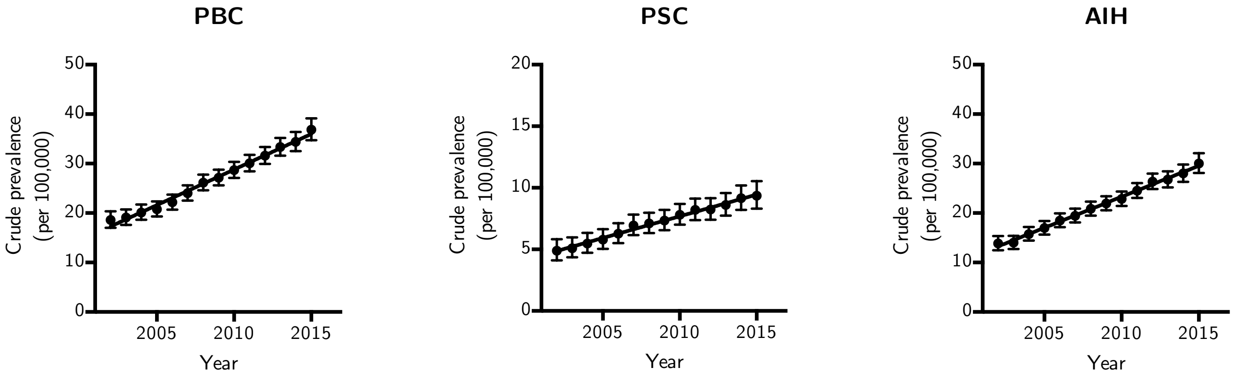

Figure S2: Crude prevalence per 100 000 over time at year end for each year of the study period. Prevalence increased over the study period for each of the autoimmune liver diseases. For PBC, there was an increase of 1.44(1.35-1.53)/100 000/year, r^2=0.991, p<0.001; PSC, there was an increase of 0.35(0.33-37)/100 000/year, r^2=0.991, p<0.001; For AIH, there was an increase of 1.26(1.20-1.31)/100 000/year, r^2=0.996, p<0.001

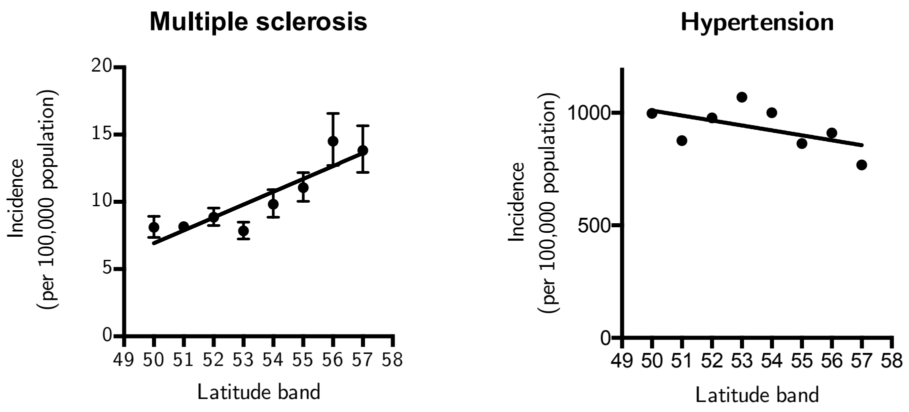

Figure S3: Incidence of multiple sclerosis and hypertension by latitude band. Figures presented are after adjustment for variations in sex, age, deprivation score, and smoking status. For multiple sclerosis, a significant positive correlation was present at 0.657(0.249 to 1.065)/100 000/degree; r^2=0.721; p=0.008. For hypertension, no significant correlation was present at −5.02(−30.27 to 20.23); r^2=0.038; p=0.644.

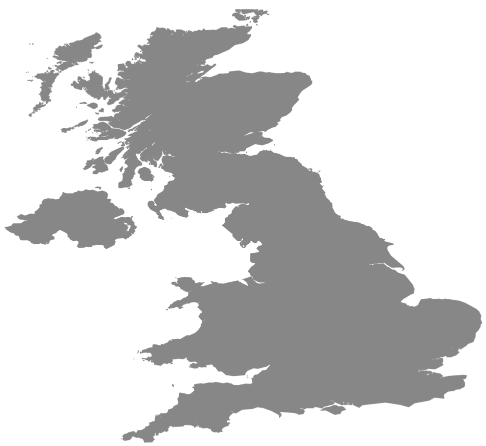

(a) Reference

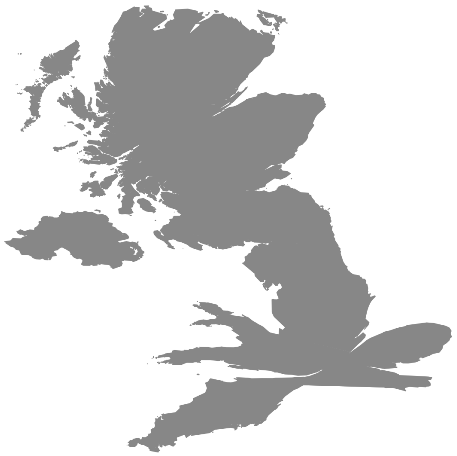

(b) PBC

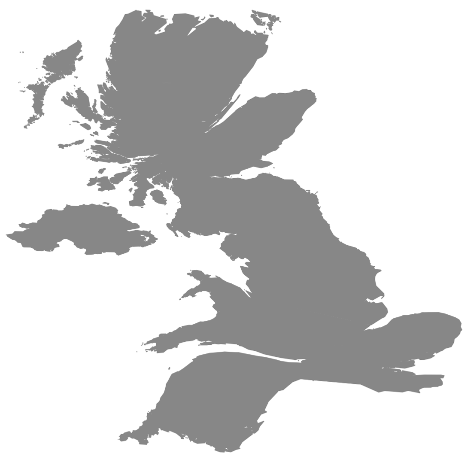

(c) PSC

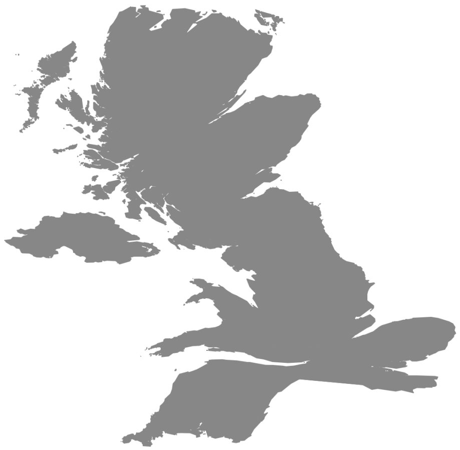

(d) AIH

Figure S4: Cartograms depicting area-proportionate resizing of latitude bands by crude incidence for autoimmune liver diseases

Supplementary Tables

Disease	Code	Description
Primary biliary cholangitis	J616000	Primary biliary cirrhosis
Primary sclerosing cholangitis	J661700	Primary sclerosing cholangitis
Autoimmune hepatitis	J661700	Chronic active hepatitis
	J614111	Autoimmune chronic active hepatitis
	J63B.00	Autoimmune hepatitis
Multiple sclerosis	F20..00	Multiple sclerosis
	F20..11	Disseminated sclerosis
	F200.00	Multiple sclerosis of the brain stem
	F201.00	Multiple sclerosis of the spinal cord
	F202.00	Generalised multiple sclerosis
	F203.00	Exacerbation of multiple sclerosis
	F204.00	Benign multiple sclerosis
	F205.00	Malignant multiple sclerosis
	F206.00	Primary progressive multiple sclerosis
	F207.00	Relapsing and remitting multiple sclerosis
	F208.00	Secondary progressive multiple sclerosis
	F20z.00	Multiple sclerosis NOS
Hypertension	G24..00	Secondary hypertension
	G240.00	Secondary malignant hypertension
	G240z00	Secondary malignant hypertension NOS
	G241.00	Secondary benign hypertension
	G241z00	Secondary benign hypertension NOS
	G244.00	Hypertension secondary to endocrine disorders
	G24z.00	Secondary hypertension NOS
	G24z000	Secondary renovascular hypertension NOS

G24zz00	Secondary hypertension NOS
G25..00	Stage 1 hypertension (NICE - Nat Ins for Hth Clin Excl 2011)
G25..11	Stage 1 hypertension
G250.00	Stage 1 hyperten (NICE 2011) without evidnce end organ damge
G251.00	Stage 1 hyperten (NICE 2011) with evidnce end organ damge
G26..00	Severe hypertension (Nat Inst for Health Clinical Ex 2011)
G26..11	Severe hypertension
G28..00	Stage 2 hypertension (NICE - Nat Ins for Hth Clin Excl 2011)
G2y..00	Other specified hypertensive disease
G2z..00	Hypertensive disease NOS
Gyu2.00	[X]Hypertensive diseases

Table S1: Diagnostic codes used in study.

		PBC incidence (/100 000/year)			PSC incidence (/100 000/year)			AIH incidence (/100 000/year)		
**Category**	**Subcategory**	**n**	**Crude**	**Adjusted**	**n**	**Crude**	**Adjusted**	**n**	**Crude**	**Adjusted**
**Population**	-	1314	2.47 (2.34-2.60)	2.47 (2.33-2.60)	396	0.74 (0.67-0.82)	0.74 (0.67-0.82)	1034	1.94 (1.83-2.06)	1.95 (1.83-2.07)
**Latitude**	50°-	111	2.16 (1.79-2.60)	1.96 (1.57-2.34)	42	0.82 (0.60-1.11)	0.76 (0.52-1.00)	103	2.00 (1.65-2.43)	1.80 (1.44-2.17)
	51°-	373	1.75 (1.58-1.94)	1.86 (1.67-2.06)	135	0.63 (0.54-0.75)	0.65 (0.54-0.75)	359	1.68 (1.52-1.87)	1.75 (1.57-1.93)
	52°-	135	1.67 (1.41-1.98)	1.75 (1.45-2.05)	68	0.84 (0.66-1.07)	0.84 (0.64-1.05)	142	1.76 (1.49-2.07)	1.76 (1.46-2.05)
	53°-	220	2.81 (2.46-3.21)	2.76 (2.40-3.13)	60	0.77 (0.59-0.99)	0.75 (0.56-0.94)	157	2.00 (1.71-2.34)	1.97 (1.66-2.28)
	54°-	161	4.49 (3.85-5.24)	4.35 (3.66-5.05)	29	0.81 (0.56-1.16)	0.80 (0.50-1.10)	79	2.20 (1.77-2.75)	2.18 (1.68-2.67)
	55°-	151	4.02 (3.43-4.72)	4.06 (3.31-4.80)	33	0.88 (0.63-1.24)	0.89 (0.56-1.22)	88	2.35 (1.90-2.89)	2.35 (1.77-2.92)
	56°-	70	4.65 (3.68-5.87)	4.19 (3.13-5.25)	9	0.60 (0.31-1.15)	0.52 (0.18-0.86)	45	2.99 (2.23-4.00)	2.45 (1.69-3.22)
	57°-	86	4.86 (3.93-6.00)	4.55 (3.52-5.58)	18	1.02 (0.64-1.61)	0.98 (0.50-1.46)	58	3.28 (2.53-4.24)	3.06 (2.18-3.93)
	Missing	7	2.37 (1.13-4.97)	2.36 (0.57-4.15)	2	0.68 (0.17-2.70)	0.56 (0.00-1.33)	3	1.01 (0.33-3.15)	0.73 (0.00-1.57)
**Sex**	Male	173	0.65 (0.56-0.76)	0.66 (0.56-0.76)	230	0.87 (0.76-0.99)	0.91 (0.79-1.03)	241	0.91 (0.80-1.03)	0.94 (0.82-1.06)
	Female	1141	4.27 (4.03-4.52)	4.24 (3.99-4.49)	166	0.62 (0.53-0.72)	0.59 (0.50-0.68)	793	2.96 (2.77-3.18)	2.92 (2.72-3.13)
**Age**	0-9.9	0	0.00 (0.00-0.00)	0.00 (0.00-0.00)	2	0.04 (0.01-0.16)	0.00 (0.00-0.00)	7	0.14 (0.06-0.28)	0.12 (0.00-0.26)
	10-19.9	1	0.02 (0.00-0.12)	0.01 (0.00-0.02)	21	0.35 (0.23-0.54)	0.03 (0.01-0.04)	55	0.92 (0.71-1.20)	0.93 (0.54-1.32)
	20-29.9	5	0.08 (0.03-0.20)	0.07 (0.01-0.13)	34	0.56 (0.40-0.78)	0.05 (0.03-0.07)	48	0.78 (0.59-1.04)	0.62 (0.44-0.81)
	30-39.9	59	0.80 (0.62-1.03)	0.71 (0.52-0.90)	28	0.38 (0.26-0.55)	0.03 (0.02-0.05)	87	1.18 (0.96-1.46)	1.10 (0.87-1.34)
	40-49.9	181	2.18 (1.88-2.52)	1.94 (1.65-2.23)	68	0.82 (0.65-1.04)	0.08 (0.06-0.10)	132	1.59 (1.34-1.88)	1.45 (1.20-1.70)
	50-59.9	318	4.34 (3.89-4.84)	4.10 (3.52-4.68)	61	0.83 (0.65-1.07)	0.08 (0.06-0.10)	221	3.02 (2.64-3.44)	2.70 (2.34-3.07)
	60-69.9	353	5.80 (5.23-6.44)	5.08 (4.53-5.63)	87	1.43 (1.16-1.76)	0.14 (0.10-0.17)	239	3.93 (3.46-4.46)	3.68 (3.13-4.23)
	70-79.9	277	6.62 (5.88-7.44)	6.22 (5.20-7.25)	65	1.55 (1.22-1.98)	0.15 (0.10-0.19)	181	4.32 (3.74-5.00)	3.72 (3.15-4.29)
	80-89.9	111	4.93 (4.10-5.94)	4.28 (3.20-5.36)	28	1.24 (0.86-1.80)	0.09 (0.05-0.13)	61	2.71 (2.11-3.48)	2.34 (1.67-3.00)
	90+	9	1.94 (1.01-3.73)	1.21 (0.38-2.05)	2	0.43 (0.11-1.72)	0.02 (0.00-0.05)	3	0.65 (0.21-2.01)	0.71 (0.00-1.65)
**Townsend deprivation quintile**	1 – least	268	2.04 (1.81-2.30)	2.21 (1.91-2.51)	99	0.75 (0.62-0.92)	0.67 (0.53-0.81)	242	1.84 (1.62-2.09)	1.85 (1.59-2.11)
	2	287	2.55 (2.27-2.87)	2.43 (2.14-2.72)	88	0.78 (0.64-0.96)	0.74 (0.58-0.91)	225	2.00 (1.76-2.28)	1.90 (1.65-2.16)
	3	282	2.59 (2.31-2.92)	2.60 (2.29-2.91)	87	0.80 (0.65-0.99)	0.81 (0.64-0.98)	219	2.01 (1.76-2.30)	2.02 (1.75-2.29)
	4	244	2.59 (2.28-2.94)	2.62 (2.28-2.96)	69	0.73 (0.58-0.93)	0.80 (0.61-1.00)	175	1.86 (1.60-2.15)	1.95 (1.65-2.25)
	5 – most	187	2.83 (2.45-3.26)	2.82 (2.35-3.28)	39	0.59 (0.43-0.81)	0.73 (0.46-0.99)	141	2.13 (1.81-2.51)	2.25 (1.82-2.67)
	Missing	46	2.32 (1.74-3.09)	2.86 (1.93-3.79)	14	0.71 (0.42-1.19)	0.75 (0.35-1.15)	32	1.61 (1.14-2.28)	1.76 (1.06-2.45)
**Smoking**	Current smoker	400	3.62 (3.28-3.99)	3.40 (3.03-3.77)	54	0.49 (0.37-0.64)	0.47 (0.33-0.61)	256	2.31 (2.05-2.62)	2.24 (1.94-2.55)
	Ex-smoker	308	4.22 (3.78-4.72)	2.93 (2.58-3.27)	69	0.95 (0.75-1.20)	0.61 (0.45-0.77)	185	2.54 (2.20-2.93)	1.98 (1.54-2.42)
	Never smoked	596	2.18 (2.01-2.36)	1.96 (1.80-2.12)	265	0.97 (0.86-1.09)	0.95 (0.83-1.07)	578	2.11 (1.95-2.29)	1.96 (1.79-2.12)
	Missing	10	0.13 (0.07-0.25)	0.87 (0.17-1.57)	8	0.11 (0.05-0.21)	0.26 (0.00-0.56)	15	0.20 (0.12-0.33)	0.30 (0.00-0.64)
**Ethnicity**	Asian	15	1.30 (0.79-2.16)	-	7	0.61 (0.29-1.28)	-	20	1.74 (1.12-2.69)	-
	Black	8	1.41 (0.71-2.83)	-	2	0.35 (0.09-1.41)	-	13	2.30 (1.33-3.95)	-
	Mixed	3	1.41 (0.45-4.37)	-	1	0.47 (0.07-3.33)	-	2	0.94 (0.23-3.75)	-
	Other	3	1.21 (0.39-3.76)	-	1	0.40 (0.06-2.87)	-	1	0.40 (0.06-2.87)	-
	White	545	2.85 (2.62-3.10)	-	163	0.85 (0.73-0.99)	-	393	2.05 (1.86-2.27)	-
	Missing	740	2.32 (2.15-2.49)	-	222	0.69 (0.61-0.79)	-	605	1.89 (1.75-2.05)	-

Table S2: Incidence of primary biliary cholangitis (PBC), primary sclerosing cholangitis (PSC), and autoimmune hepatitis (AIH) for the time period 2002-01-01 to 2016-05-10. 'Adjusted' figures are adjusted for one or all of latitude, sex, age, Townsend deprivation quintile, and smoking status by direct standardisation as appropriate. Figures are per 100 000 population/year with 95% confidence intervals.

Year	PBC n	PBC incidence	PSC n	PSC incidence	AIH n	AIH incidence
2002	68	2.64 (2.08-3.35)	19	0.74 (0.47-1.16)	34	1.32 (0.94-1.85)
2003	73	2.39 (1.90-3.01)	25	0.82 (0.55-1.21)	50	1.64 (1.24-2.16)
2004	74	2.27 (1.81-2.85)	26	0.80 (0.54-1.17)	77	2.36 (1.89-2.96)
2005	78	2.20 (1.76-2.75)	25	0.71 (0.48-1.04)	76	2.15 (1.71-2.69)
2006	100	2.68 (2.21-3.26)	28	0.75 (0.52-1.09)	72	1.93 (1.53-2.43)
2007	95	2.47 (2.02-3.02)	28	0.73 (0.50-1.05)	66	1.71 (1.35-2.18)
2008	95	2.35 (1.92-2.87)	26	0.64 (0.44-0.94)	59	1.46 (1.13-1.88)
2009	109	2.57 (2.13-3.10)	35	0.83 (0.59-1.15)	76	1.79 (1.43-2.24)
2010	107	2.54 (2.10-3.07)	31	0.74 (0.52-1.05)	82	1.95 (1.57-2.42)
2011	105	2.49 (2.06-3.01)	35	0.83 (0.60-1.15)	98	2.32 (1.91-2.83)
2012	108	2.53 (2.10-3.06)	24	0.56 (0.38-0.84)	101	2.37 (1.95-2.88)
2013	112	2.70 (2.25-3.25)	29	0.70 (0.49-1.01)	91	2.20 (1.79-2.70)
2014	90	2.35 (1.91-2.89)	33	0.86 (0.61-1.21)	58	1.51 (1.17-1.96)
2015	80	2.43 (1.95-3.03)	25	0.76 (0.51-1.12)	75	2.28 (1.82-2.86)

Table S3: Incidence over time. Incident cases of autoimmune liver diseases are given per 100 000/year

Category	Subcategory	PBC prevalence (/100 000) n	PBC Crude	PBC Adjusted	PSC prevalence (/100 000) n	PSC Crude	PSC Adjusted	AIH prevalence (/100 000) n	AIH Crude	AIH Adjusted
**Population**	-	1299	39.62 (37.52-41.83)	39.62 (37.50-41.74)	353	10.77 (9.70-11.95)	10.77 (9.65-11.88)	1116	34.04 (32.10-36.10)	34.04 (32.06-36.02)
**Latitude**	50°-	83	33.45 (26.97-41.47)	28.16 (21.88-34.44)	27	10.88 (7.46-15.86)	9.71 (5.87-13.55)	93	37.47 (30.58-45.92)	32.19 (25.38-39.00)
	51°-	381	28.50 (25.78-31.51)	30.59 (27.50-33.69)	141	10.55 (8.94-12.44)	10.61 (8.84-12.38)	382	28.57 (25.85-31.59)	29.78 (26.76-32.79)
	52°-	84	27.37 (22.10-33.90)	27.29 (21.44-33.15)	30	9.77 (6.83-13.98)	9.90 (6.29-13.50)	88	28.67 (23.27-35.34)	29.00 (22.89-35.10)
	53°-	161	43.34 (37.13-50.58)	42.01 (35.51-48.51)	34	9.15 (6.54-12.81)	8.55 (5.64-11.45)	143	38.49 (32.67-45.35)	37.20 (31.04-43.37)
	54°-	153	64.94 (55.43-76.09)	63.86 (53.70-74.02)	27	11.46 (7.86-16.71)	11.41 (7.09-15.74)	98	41.60 (34.13-50.71)	42.69 (34.10-51.30)
	55°-	195	49.09 (42.66-56.48)	49.79 (42.02-57.56)	40	10.07 (7.39-13.73)	10.46 (7.01-13.91)	148	37.25 (31.71-43.77)	40.47 (33.02-47.93)
	56°-	103	66.37 (54.71-80.51)	60.95 (48.53-73.36)	18	11.60 (7.31-18.41)	10.63 (5.67-15.58)	71	45.75 (36.25-57.73)	41.00 (31.17-50.82)
	57°-	129	64.70 (54.44-76.88)	61.08 (50.11-72.05)	34	17.05 (12.18-23.87)	16.70 (10.77-22.63)	88	44.14 (35.81-54.39)	39.11 (30.44-47.78)
		10	36.04 (19.39-66.99)	37.40 (15.06-59.74)	2	7.21 (1.80-28.82)	8.51 (0.00-19.85)	5	18.02 (7.50-43.30)	18.50 (3.98-33.03)
**Sex**	Male	143	8.80 (7.47-10.36)	8.82 (7.37-10.28)	205	12.61 (11.00-14.46)	13.25 (11.43-15.07)	283	17.41 (15.50-19.56)	17.93 (15.83-20.04)
	Female	1156	69.92 (66.00-74.07)	69.35 (65.36-73.33)	148	8.95 (7.62-10.52)	8.69 (7.28-10.09)	833	50.38 (47.08-53.93)	50.36 (46.92-53.79)
**Age**	0-9.9	0	0.00 (0.00-0.00)	0.00 (0.00-0.00)	1	0.30 (0.04-2.15)	0.06 (0.00-0.18)	2	0.61 (0.15-2.42)	2.55 (0.00-7.43)
	10-19.9	0	0.00 (0.00-0.00)	0.00 (0.00-0.00)	11	2.95 (1.64-5.33)	3.64 (1.40-5.89)	28	7.52 (5.19-10.89)	9.02 (4.34-13.68)
	20-29.9	2	0.52 (0.13-2.08)	0.77 (0.00-1.95)	33	8.60 (6.11-12.10)	7.23 (4.14-10.32)	67	17.46 (13.74-22.18)	16.74 (10.76-22.71)
	30-39.9	31	7.37 (5.18-10.48)	5.88 (3.78-7.98)	42	9.98 (7.38-13.51)	8.30 (5.72-10.88)	82	19.49 (15.70-24.20)	23.58 (14.76-32.40)
	40-49.9	121	25.33 (21.20-30.27)	21.04 (17.27-24.82)	44	9.21 (6.86-12.38)	9.09 (5.44-12.73)	132	27.64 (23.30-32.78)	28.08 (18.51-37.65)
	50-59.9	260	55.21 (48.89-62.35)	52.81 (41.85-63.77)	67	14.23 (11.20-18.08)	12.34 (9.36-15.32)	201	42.68 (37.17-49.01)	35.66 (30.68-40.65)
	60-69.9	363	93.82 (84.65-103.98)	84.44 (70.06-98.82)	73	18.87 (15.00-23.73)	15.81 (12.07-19.55)	279	72.11 (64.12-81.09)	68.96 (55.14-82.77)
	70-79.9	344	129.23 (116.27-143.63)	100.97 (89.93-112.02)	54	20.29 (15.54-26.49)	19.14 (12.56-25.72)	221	83.02 (72.77-94.72)	66.06 (56.93-75.20)
	80-89.9	163	117.21 (100.53-136.66)	84.97 (70.48-99.46)	26	18.70 (12.73-27.46)	12.41 (7.34-17.49)	97	69.75 (57.16-85.11)	57.29 (44.49-70.10)
	90+	15	48.39 (29.17-80.27)	29.64 (13.90-45.37)	2	6.45 (1.61-25.80)	3.38 (0.00-7.90)	7	22.58 (10.77-47.37)	33.83 (0.96-66.69)
**Townsend deprivation quintile**	1 – least	269	36.43 (32.33-41.05)	38.15 (32.99-43.30)	13	10.23 (5.94-17.61)	9.84 (6.14-13.53)	233	31.56 (27.75-35.88)	31.40 (26.79-36.00)
	2	295	43.15 (38.50-48.36)	38.99 (34.48-43.51)	88	11.92 (9.67-14.69)	12.77 (5.17-20.37)	261	38.18 (33.81-43.10)	35.87 (31.40-40.34)
	3	268	39.24 (34.82-44.24)	38.92 (34.29-43.56)	83	12.14 (9.79-15.05)	10.33 (8.03-12.64)	247	36.17 (31.93-40.97)	36.30 (31.77-40.83)
	4	256	41.82 (37.00-47.27)	42.38 (37.10-47.66)	70	10.25 (8.11-12.96)	11.07 (8.57-13.56)	189	30.87 (26.77-35.61)	32.09 (27.41-36.78)
	5 – most	175	40.28 (34.74-46.72)	42.09 (34.83-49.35)	64	10.45 (8.18-13.36)	10.19 (7.80-12.58)	156	35.91 (30.69-42.01)	37.31 (30.60-44.00)
	Missing	36	28.32 (20.43-39.27)	34.23 (22.82-45.63)	35	8.06 (5.78-11.22)	11.26 (8.44-14.08)	30	23.60 (16.50-33.76)	27.27 (16.52-38.03)
**Smoking**	current smoker	347	55.50 (49.96-61.66)	48.60 (43.04-54.15)	41	6.56 (4.83-8.91)	5.27 (3.54-7.00)	273	43.67 (38.78-49.17)	40.01 (34.37-45.66)
	ex-smoker	351	78.66 (70.85-87.34)	52.55 (46.96-58.13)	60	13.45 (10.44-17.32)	8.49 (6.19-10.80)	234	52.44 (46.14-59.61)	36.33 (31.46-41.21)
	never smoked	596	36.99 (34.13-40.08)	31.43 (28.89-33.97)	247	15.33 (13.53-17.36)	14.13 (12.34-15.93)	595	36.92 (34.07-40.01)	32.73 (29.91-35.57)
	Missing	5	0.84 (0.35-2.02)	10.69 (0.61-20.77)	5	0.84 (0.35-2.02)	3.92 (0.00-8.14)	14	2.35 (1.39-3.97)	23.31 (8.09-38.55)
**Ethnicity**	Asian	13	15.34 (8.91-26.41)	-	9	10.62 (5.53-20.41)	-	17	20.06 (12.47-32.26)	-
	Black	5	11.31 (4.71-27.16)	-	4	9.04 (3.39-24.10)	-	16	36.18 (22.16-59.05)	-
	Mixed	2	10.80 (2.70-43.19)	-	1	5.40 (0.76-38.34)	-	3	16.20 (5.23-50.24)	-
	Other	2	11.36 (2.84-45.41)	-	2	11.36 (2.84-45.41)	-	4	22.72 (8.53-60.52)	-
	White	590	41.37 (38.16-44.85)	-	141	9.89 (8.38-11.66)	-	502	35.20 (32.25-38.42)	-
	Missing	687	40.71 (37.78-43.87)	-	196	11.62 (10.10-13.36)	-	574	34.02 (31.34-36.92)	-

Table S4: Prevalence of primary biliary cholangitis (PBC), primary sclerosing cholangitis (PSC), and autoimmune hepatitis (AIH) for 2015. 'Adjusted' figures are adjusted for one or all of latitude, sex, age, Townsend deprivation quintile, and smoking status by direct standardisation as appropriate. Figures are per 100 000 population with 95% confidence intervals.

**Latitude**	**MS n**	**MS incidence**	**MS adj. incidnce**	**HTN n**	**HTN incidence**	**HTN adj incidence**
50°-	416	8.11 (7.37-8.93)	8.24 (7.40-9.07)	43003	997.75 (988.37-1,007.23)	830.12 (821.90-838.35)
51°-	1734	8.15 (7.78-8.54)	8.12 (7.73-8.50)	161303	876.25 (871.98-880.54)	928.56 (924.06-933.06)
52°-	714	8.86 (8.24-9.54)	8.90 (8.23-9.56)	67525	977.72 (970.37-985.12)	980.83 (973.43-988.24)
53°-	613	7.84 (7.24-8.49)	7.82 (7.19-8.44)	71343	1,069.49 (1,061.67-1,077.36)	1,053.76 (1,046.09-1,061.42)
54°-	352	9.83 (8.86-10.92)	10.35 (9.24-11.46)	30383	1,000.43 (989.24-1,011.74)	1,013.13 (1,001.58-1,024.68)
55°-	414	11.06 (10.04-12.17)	11.45 (10.21-12.69)	27812	863.80 (853.71-874.01)	902.11 (889.86-914.36)
56°-	218	14.51 (12.71-16.57)	14.73 (12.62-16.84)	11557	910.42 (893.97-927.17)	851.28 (834.32-868.23)
57°-	244	13.82 (12.19-15.67)	13.67 (11.86-15.48)	11619	768.37 (754.53-782.47)	710.22 (696.41-724.02)
Missing	19	6.44 (4.10-10.09)	6.63 (3.43-9.83)	2084	824.58 (789.92-860.75)	874.39 (832.29-916.49)

Table S5: Incidence of multiple sclerosis and hypertension by latitude band.
Incident cases are presented per 100 000/year

**Latitude**	**PBC**	**PSC**	**AIH**
50	65 (56-74)	62 (40-68)	62 (50-72)
51	62 (53-73)	52.5 (40.5-68)	58 (43-69)
52	63 (53-75)	60 (48-70)	60 (48-69)
53	63 (52.5-71)	59.5 (48.5-68.5)	56 (41-67)
54	62 (52-72)	45 (32-66)	58 (46-68)
55	59 (51-68)	51 (25-66)	58 (41-70)
56	65 (52-74)	45 (40-64)	54 (41-64)
57	59 (51-68)	63.5 (47-77)	55 (44-67)

Table S6: Age at disease incidence 2002-2016 by latitude band. Figures denote median age and interquartile range. For PBC, median age of incidence did not correlate with latitude at -0.48(-1.29-0.34) years/degree, r^2=0.254, p=0.203; For PSC, there was no significant change with latitude: -0.82(-3.74-2.11) years/degree, r^2=0.072, p=0.521; For AIH, incident cases were younger by -0.87(-1.489 to -0.25) years/degree, r^2=0.663, p=0.014

Latitude	PBC men	PBC women	PSC men	PSC women	AIH men	AIH women
50°-	14	97	22	20	21	82
51°-	46	327	73	62	87	272
52°-	19	116	38	30	39	103
53°-	28	192	39	21	27	130
54°-	23	138	18	11	22	57
55°-	16	135	20	13	23	65
56°-	10	60	5	4	12	33
57°-	15	71	14	4	8	50
Missing	2	5	1	1	2	1
Chi-squared p		0.894		0.547		0.196

Table S7: Sex distribution of incident diagnoses 2002-2016 by latitude band. The overall ratios of women:men were 6.6 for PBC, 0.7:1 for PSC, and 3.3:1 for AIH.

Year	PBC men	PBC women	PSC men	PSC women	AIH men	AIH women
2002	12	56	4	30	4	30
2003	8	65	8	42	8	42
2004	7	67	20	57	20	57
2005	15	63	13	63	13	63
2006	14	86	21	51	21	51
2007	14	81	16	50	16	50
2008	7	88	16	43	16	43
2009	17	92	19	57	19	57
2010	11	96	23	59	23	59
2011	15	90	26	72	26	72
2012	13	95	17	84	17	84
2013	17	95	18	73	18	73
2014	10	80	13	45	13	45
2015	8	72	20	55	20	55
2016	5	15	18	7	7	12
p-value		0.673		0.879		0.341

Table S8: Sex ratios of incident cases over time. Chi-sq test for trend

Year	PBC	PSC	AIH
2002	66.5 (53.5-73)	57 (45-68)	52 (37-64)
2003	60 (52-69)	48 (39-62)	52.5 (35.5-68)
2004	61 (52-70)	51 (40-61)	54 (43-62)
2005	61 (53-73)	58.5 (39.5-67.5)	59 (40-72)
2006	60 (50-70)	46 (30-62)	52 (34-63)
2007	60 (52-70)	51.5 (43-61)	57 (40-69)
2008	60 (51-68)	53 (37-62)	54 (41-69)
2009	62 (49-71)	51 (29-67)	55 (31-67)
2010	64 (53-74)	56.5 (38-68.5)	56.5 (44.5-68)
2011	60 (51.5-71.5)	55 (25-70)	56 (40-67)
2012	62 (52-68)	48.5 (28-73)	59 (47.5-66)
2013	60.5 (52-71)	60.5 (45-68)	56.5 (42-68)
2014	59 (51-70)	60 (41-74)	58.5 (40-70)
2015	63 (49-71)	62 (55-69)	58.5 (38-66.5)

Table S9: Age at disease incidence 2002-2016 by year of diagnosis for PBC, PSC, and AIH. Figures denote median age and interquartile range. For PBC, there was no significant change over time ($-0.09(-0.39$ to 0.21) years/year; r^2=0.033, p=0.532). For PSC, there was no significant change over time (0.59(-0.06 to 1.26) years/year; r^2=0.241, p=0.074). For AIH, there was a significant increase in age of incidence over time (0.42(0.15 to 0.70) years/year; r^2=0.483, p=0.006).

Latitude band	Townsend deprivation quintile						
	**1**	**2**	**3**	**4**	**5**	**Missing**	**Total**
**50**	235 796	206 805	181 580	146 256	73 019	32 879	876 335
**%**	26.91	23.6	20.72	16.69	8.33	3.75	100
**51**	820 212	686 246	822 839	755 954	453 676	243 339	3 782 266
**%**	21.69	18.14	21.76	19.99	11.99	6.43	100
**52**	353 140	266 288	233 751	226 977	148 738	86 967	1 315 861
**%**	26.84	20.24	17.76	17.25	11.3	6.61	100
**53**	288 799	242 281	231 968	201 139	180 421	71 600	1 216 208
**%**	23.75	19.92	19.07	16.54	14.83	5.89	100
**54**	91 075	81 580	91 143	101 576	100 439	20 130	485 943
**%**	18.74	16.79	18.76	20.9	20.67	4.14	100
**55**	67 669	104 149	113 372	136 385	170 032	54 708	646 315
**%**	10.47	16.11	17.54	21.1	26.31	8.46	100
**56**	27 775	52 222	52 368	54 572	55 600	11 995	254 532
**%**	10.91	20.52	20.57	21.44	21.84	4.71	100
**57 and 58**	41 882	89 917	75 210	56 845	35 344	21 299	320 497
**%**	13.07	28.06	23.47	17.74	11.03	6.65	100
**Missing**	4614	8278	13 981	14 330	12 326	3555	57 084
**%**	8.08	14.5	24.49	25.1	21.59	6.23	100
**Total**	1 930 962	1 737 766	1 816 212	1 694 034	1 229 595	546 472	8 955 041
**%**	21.56	19.41	20.28	18.92	13.73	6.1	100

Table S10: Deprivation score by latitude band.
